# Draft Genome Sequence of *Methanohalophilus mahii* Strain DAL1 Reconstructed from a Hydraulic Fracturing-Produced Water Metagenome

**DOI:** 10.1128/genomeA.00899-16

**Published:** 2016-09-01

**Authors:** Daniel Lipus, Amit Vikram, Daniel E. Ross, Kyle Bibby

**Affiliations:** a Department of Civil and Environmental Engineering, University of Pittsburgh, Pittsburgh, Pennsylvania, USA; b Department of Computational and Systems Biology, University of Pittsburgh, Pittsburgh, Pennsylvania, USA; c National Energy Technology Laboratory (NETL), Pittsburgh, Pennsylvania, USA; d AECOM, Pittsburgh, Pennsylvania, USA

## Abstract

We report here the 1,882,100-bp draft genome sequence of *Methanohalophilus mahii* strain DAL1, recovered from Marcellus Shale hydraulic fracturing-produced water using metagenomic contig binning. Genome annotation revealed several key methanogenesis genes and provides valuable information on archaeal activity associated with hydraulic fracturing-produced water environments.

## GENOME ANNOUNCEMENT

The Marcellus Shale is the largest natural gas reservoir in the United States and is a leading producer of methane through high-volume hydraulic fracturing (1, 2). While the methane in the Marcellus Shale is assumed to be of thermogenic origin, recent observations suggest biogenic methanogenesis potentially contributes to methane production in the Marcellus Shale during the fracturing process (3–5). Several studies have investigated overall microbial populations associated with Marcellus Shale or similar hydraulic fracturing operations (6–8), with *Archaea* being detected at low quantities (6, 9, 10). At this point, little is known about the activity of methanogens in hydraulic fracturing environments.

Here, we present the draft genome sequence of *Methanohalophilus mahii* strain DAL1, recovered from the metagenome of Marcellus Shale hydraulic fracturing-produced water. Sequencing libraries were prepared using Illumina Nextera XT and sequenced using Illumina MiSeq technology (Illumina, San Diego, CA). Sequencing reads were quality trimmed (Q30) and *de novo* assembled into contigs using CLC Genomics Workbench version 8.5.1 (CLC Bio, Aarhus, Denmark) and SPAdes version 3.5.1 (11). Assembled contigs were grouped into genome bins with MaxBin (12) and Vizbin (13) and taxonomy assessed with PhyloPythia (14). Metagenomic reads were mapped against binned contigs and reassembled using SPAdes.

The final draft genome contained 58 contigs of 5,000 bp to 131,826 bp in length and an *N*_50_ of 52,299 bp. The total genome size was 1,894,170 bp, with a mean G+C content of 42.4% and an average of 90-fold coverage. Draft genome completeness and contamination were estimated using CheckM (15). The final draft genome was found to be 93.9% complete and contain 1.1% contamination.

The draft genome was annotated by Rapid Annotations using Subsystems Technology (RAST) (16, 17), revealing 2,066 gene-coding sequences (CDSs) and 49 RNA sequences (46 tRNA, 16S, 23S, and 5S rRNA). Extraction and phylogenetic analysis of the 16S rRNA gene sequence using RDP (18) and CLC Bio suggested *Methanohalophilus mahii* strain DAL1 to be closely related to *Methanohalophilus mahii* strain DSM 5219 (99% BLASTn [19] nucleotide identity). RAST (16, 17) and KEGG (20) annotation allowed the discovery of all core methanogenesis enzymes necessary for the conversion of CO_2_ to methane, including the heterodisulfide reductase (*hdr*), methyl coenzyme M reductase (*mcr*), and methenyl-tetrahydromethanopterin cyclohydrolase (*mch*). Moreover, the coenzyme M methyltransferase (*mtbA*) and the trimethylamine methyltransferase (*mttB*) genes were identified. These observations suggest *Methanohalophilus mahii* strain DAL1 to have the genetic potential for hydrogenotrophic (CO_2_ to methane) and methyl reduction (trimethylamine to methane) methanogenesis (21).

Annotation also revealed the potassium uptake proteins TrkA and TrkH and the osmoprotectant transporter ProP. These findings agree with previous observations that members of the genus *Methanohalophilus* accumulate potassium ions and uptake glycine and betaine in response to osmotic stress (22–24).

The analysis of the *Methanohalophilus mahii* sp. DAL1 draft genome indicates the potential for methanogenic activity associated with Marcellus Shale hydraulic fracturing operations.

### Accession number(s).

This whole-genome shotgun project has been deposited at DDBJ/ENA/GenBank under the accession no. MAFX00000000. The version described in this paper is version MAFX01000000.
